# Impact of shift work on surgical outcomes at different times in patients with acute type A aortic dissection: A retrospective cohort study

**DOI:** 10.3389/fcvm.2022.1000619

**Published:** 2022-10-28

**Authors:** Xiang Zhang, Weiwei Lv, Xin Liu, Kai Liu, Shaozhong Yang

**Affiliations:** ^1^Department of Anesthesiology, Qilu Hospital of Shandong University, Jinan, Shandong, China; ^2^Department of Radiology, Qilu Hospital of Shandong University, Jinan, Shandong, China; ^3^Department of Cardiovascular Surgery, Qilu Hospital of Shandong University, Jinan, Shandong, China

**Keywords:** acute type A aortic dissection, total arch replacement, shift work, off-hours, hospital mortality

## Abstract

**Background:**

To investigate the effect of shift work on surgical outcomes at different times in patients with acute type A aortic dissection (ATAAD).

**Materials and methods:**

Patients with ATAAD who underwent total arch replacement at Qilu Hospital of Shandong University from January 2015 to March 2022 were retrospectively analyzed. All patients were managed according to the green channel emergency management strategy, and a professional cardiac team was arranged during off-hours. Based on surgery time and symptom onset to procedure time, the patients were divided into weekday, weekend and holiday groups; daytime and nighttime groups; intervention time ≤48 h and >48 h groups; working hours and off-hours groups. In-hospital mortality between these groups was compared.

**Results:**

In total, 499 ATAAD patients underwent surgery within 7 days of symptom onset, and the in-hospital mortality rate was 10% (*n* = 50/499). Among the 499 patients, 320 (64.13%), 128 (25.65%) and 51 (10.22%) underwent surgery on weekdays, weekends and holidays, respectively. In-hospital mortality and 7-day mortality showed no significant difference among the three groups. Two hundred twenty-seven (45.5%) underwent daytime surgery, and 272 (54.5%) underwent nighttime surgery. Durations of ICU stay and hospital stay were significantly different between the two groups (*P* < 0.05). There was no significant differences in in-hospital mortality (9.2% vs. 10.7%) and 7-day mortality (4.4% vs. 6.6%). 221 patients (44.3%) and 278 patients (55.7%) were included in the intervention time ≤48 h and >48 h groups, respectively. Acute renal injury, ICU stay and hospital stay were significantly different (*P* < 0.05) whereas 7-day mortality (5.0% vs. 6.1%) and in-hospital mortality (8.6% vs. 11.1%) were not. Furthermore, 7-day (1.9% vs. 6.6%) and in-hospital mortality (11.1% vs. 9.8%) showed no difference between working hours group (*n* = 108) and off-hours group (*n* = 391). Cox regression analysis showed that postoperative acute renal injury (HR = 2.423; 95% CI, 1.214–4.834; *P* = 0.012), pneumonia (HR = 2.542; 95% CI, 1.186–5450; *P* = 0.016) and multiple organ dysfunction (HR = 11.200; 95% CI, 5.549–22.605; *P* = 0.001) were the main factors affecting hospital death in ATAAD patients.

**Conclusion:**

Under the management of a professional cardiac surgery team with dedicated off-hours shifts, surgery time was not related to in-hospital mortality in ATAAD patients.

## Introduction

Acute type A aortic dissection (ATAAD) is a life-threatening emergency with high mortality (1). The death risk of ATAAD was estimated to be 1 to 2% per hour, and non-surgical treatment was associated with mortality in nearly 60% of patients (2). Emergency open ascending aorta replacement is the preferred method to prevent adverse outcomes (3, 4). However, such operations rely more on senior surgical teams with extensive experience in dealing with complex vascular patients (5). Affected by many factors, ATAAD surgery usually occurs at off-hours. It is well known that the allocation and ability of hospital medical staff at night, weekends and holidays may be different compared with weekdays, and the attention of medical staff is also affected, which may affect the perioperative outcome of ATAAD patients.

Nighttime surgery can lead to fatigue and inattention among doctors and nurses (6). Studies have found that nighttime and weekend surgery can increase the in-hospital mortality of ATAAD patients (7, 8). However, the recent results of the international registry of artic dissection (IRAD) show that there was no difference in mortality between the daytime and nighttime, workday and weekend cohorts of ATAAD patients (9). Another study also confirmed that there was no relationship between the 30-day mortality of ATAAD patients and whether the operation was performed on weekends or weekdays (10). However, a meta-analysis found that weekend admission or surgery for acute aortic dissection may be associated with increased mortality (11). A recent study showed that the mortality of patients with acute aortic dissection admitted on weekends was higher than that of patients admitted on weekdays, while there was no difference between holidays and working days (12). The difference in the above results may be related to the different allocations of medical personnel and resources in different medical units during off-hours.

To reduce the impact of weekend and holiday effects on the survival rate of surgical patients, Qilu Hospital of Shandong University has established an ATAAD operation team composed of cardiac surgeons, anesthesiologists, and operating room nurses since 2015 and has arranged special shift personnel at night, weekends and holidays. All patients were treated with an emergency green channel integrated management strategy. Cardiac surgeons and anesthesiologists on the team are required to have experience in more than 50 cases of aortic dissection. To avoid excessive fatigue, all operations were performed by the team in a shift work mode. The purpose of this study was to investigate whether there were differences in the in-hospital mortality of patients undergoing ATAAD surgery in our center on weekdays, weekends or holidays, daytime and nighttime under the shift work mode.

## Patients and methods

The STROBE guidelines were used to ensure the reporting of this study.

### Study design and participants

This single-center retrospective cohort study was conducted in Qilu Hospital, Shandong University, Jinan, China, using data from the database. This study was carried out in accordance with the Declaration of Helsinki (2013 Edition) and was approved by Qilu Hospital of Shandong University Medical Ethics Committee (No. KYLL-202204-048). From January 2015 to March 2022, the data of patients who were diagnosed with ATAAD and underwent total arch replacement in our center were collected from the database. Because of the retrospective nature of the study, any requirement for informed consent was waived.

The green channel emergency management strategy consisted of 3 parts. (1) First, emergency procedures related to ATAAD were formulated, and a special green channel was established for the patients, including pre-hospital and intra-hospital first aid. Printing the green channel sign of aortic dissection and hanging warning lights to help ensure the patients were sent to the designated area quickly and accurately. (2) Second, the hospital departments including emergency department, cardiac surgery department, laboratory department, imaging department, anesthesia department and operating room were combined together. A special rescue team composed of personnel with sufficient work experience has been established. The work responsibilities of the medical staff in each department were the comprehensive management strategy of emergency green channel, and regular training and evaluation were conducted. (3) The emergency physicians were responsible for quickly identifying patients’ diseases and evaluating the severity of their conditions. In addition, they were responsible for arranging the medical guideline to send patients to the designated doctor’s office for diagnosis and emergency treatment. Therefore, in our center, regardless of whether the patients are admitted to the hospital on weekdays, weekends or holidays, once the patients are diagnosed, they are prepared for emergency surgical treatment. According to the standard of diagnosis and treatment, all patients were sedated and were given analgesics before the operation, and their blood pressure (100–120 mmHg) and heart rate (60–80 beats/min) were strictly controlled.

**FIGURE 1:**
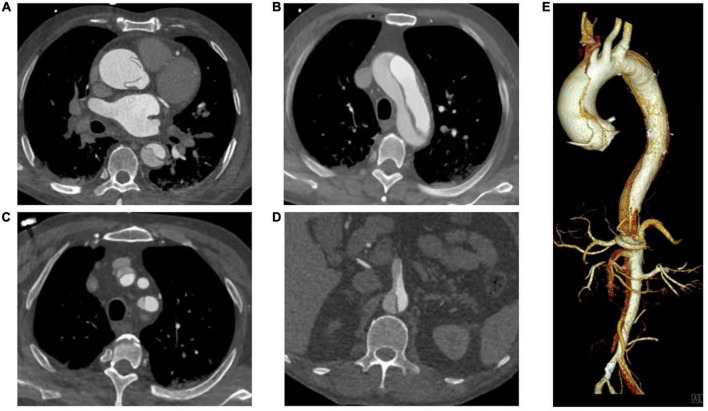
CT Angiography of the Aorta. **(A)** Ascending aorta; **(B)** Aortic arch; **(C)** Branch of aortic arch (involving brachiocephalic trunk and left subclavian artery); **(D)** Aortic dissection involving superior mesenteric artery; **(E)** Volumetric CT scanning: 3D reconstruction.

In recent decades, ATAAD has generally been defined as any disease involving the ascending aorta within 14 days after the onset of symptoms (4). Since the time delay from symptom onset to surgical treatment in ATAAD patients significantly changes the survival rate (7), this study selected patients within 7 days of symptom onset according to the new aortic dissection time classification proposed by the IRAD (13). The inclusion criteria were patients aged 18–80 years, diagnosed with ATAAD (CT angiography of the Aorta, Figure 1) and undergoing total aortic arch replacement. The primary exclusion criteria were conservative treatment, death before or during surgery, and symptom onset > 7 days.

### Surgical techniques

Surgeries were a standard longitudinal median sternotomy. The procedure was mostly performed with right axillary artery cannulation for cardiopulmonary bypass (CPB) and selective antegrade cerebral perfusion under moderate hypothermic circulatory arrest at approximately 26–27°C. A total arch replacement using a tetrafurcated graft with implantation of a stented graft in the descending aorta. Related surgical techniques have been described in detail in previously publications of our center (14).

We set up two special data reviewers for the study, consisting of a cardiac surgeon and a radiologist, who performed a consistent ATAAD diagnostic code and selected patients strictly according to the inclusion and exclusion criteria.

Unlike other studies, which grouped patients according to the date of admission (8, 9, 12), we divided patients into the following three groups according to the surgery date and the Chinese legal holidays of the current year: weekdays, weekends and holidays. Based on the data of the patients operated on weekdays, the mortality risk of patients operated on during weekends and holidays was compared.

The patients were divided into daytime and nighttime groups according to the start and end times of the operation. Daytime is defined as 8:00–17:00, and nighttime is defined as 17:00 to 8:00. If the start time and end time belong to different time ranges, a time range of 50% of the operation time should be considered. In addition, according to the time from symptom onset to surgical intervention, the patients were divided into ≤48 h and >48 h groups. We also divided the operation into working hours group and off-hours group according to whether the operation was performed during working hours, and an intergroup analysis was conducted.

### Data collection

Patient data, including age, sex, time from symptom onset to admission, preoperative laboratory examination, time from admission to operation, operation date, operation start and end time; CPB time, intraoperative blood transfusion, postoperative ICU stay, postoperative complications, hospital stay, hospitalization outcome, etc., were collected.

### Outcomes

The primary outcome was in-hospital mortality. The secondary outcomes were 7-day mortality, 30-day mortality, postoperative complications and hospitalization outcome, etc.

### Sample size calculation

The study sample size was calculated with PASS 15 (NCSS, LLC. Kaysville, Utah, USA). According to previous studies in China and the IRAD (6, 10), the mortality rates of the daytime group and the nighttime group were 5.84% and 15.33%, respectively, and there was no difference between the working day group and the weekend group. Therefore, a total of 219 patients per group provided 90% power at a 2-sided α of 5%. Assuming a 5% dropout rate, a total of at least 462 patients are needed.

### Statistical analysis

SPSS 24.0 statistical software was used for statistical analysis. All statistical tests of hypotheses were two-sided and performed at the 0.05 level of significance. The missing values of intraoperative blood transfusion were supplemented by the mean value substitution method. Quantitative variables were expressed as the mean and standard deviation (SD) or the median and interquartile range (IQR) for non-normally distributed data, and qualitative variables were expressed as frequencies and percentages. For continuous data, if the measured data conformed to a normal distribution, analysis of variance was used; when the data did not conform to a normal distribution, the Kruskal–Wallis test was used. For categorical variables, the Chi-square test or Fisher’s exact test was used for comparison. Kaplan-Meier analysis was used to estimate the survival function of patients who survived during hospitalization, while the log-rank test was used for comparison. Univariate and multivariate Cox regression analyses were used to evaluate the correlation between surgical hospitalization results at different times and the related risk factors for in-hospital death, and HRs and 95% CIs were calculated. The variables with *P* < 0.1 in univariate analysis were included in multivariate regression analysis.

## Results

**FIGURE 2:**
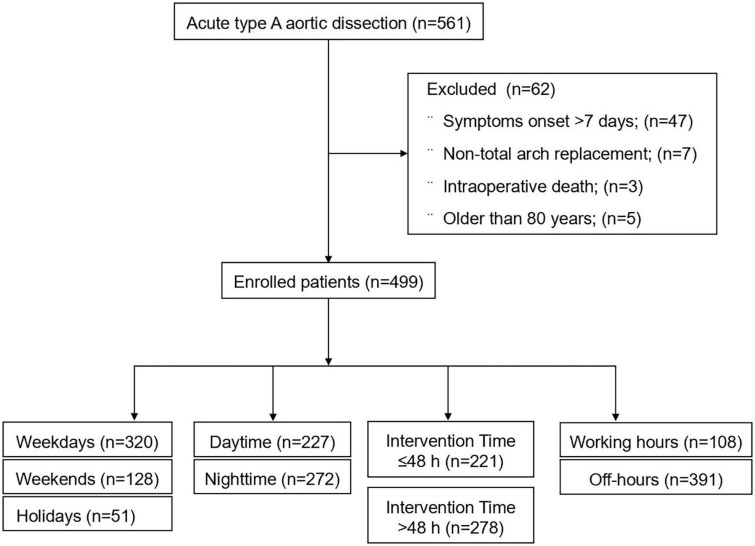
Study flow diagram.

From January 2015 to March 2022, a total of 561 patients with ATAAD received surgical treatment, including 47 patients with the onset of symptoms for more than 7 days, 5 patients over the age of 80 years, 3 patients who died during the operation, and 7 patients who only underwent ascending aortic replacement (Figure 2). Finally, a total of 499 patients with ATAAD who underwent surgery were included in our study, with an average age of 52.79 ± 11.88 years, of which 70.54% were male (*n* = 352/499), the in-hospital mortality rate was 10.0% (*n* = 50/499), and the 7-day mortality rate was 5.6% (*n* = 28/499). The most frequent comorbidity was hypertension [77.76% (*n* = 388/499)] (Table 1).

**TABLE 1:** Preoperative patient characteristics.

Characteristic	Total cohort (*n* = 499)	Weekday (*n* = 320)	Weekend (*n* = 128)	Holiday (*n* = 51)	*P*-value
Age (years)	52.79 (11.88)	52.56 (11.94)	53.73 (11.90)	53.90 (11.69)	0.549
Sex (male)	352 (70.54)	229 (71.6)	92 (71.9)	31 (60.8)	0.272
Hypertension	388 (77.76)	249 (77.8)	95 (74.2)	44 (86.3)	0.216
Coronary disease	52 (10.42)	34(10.6)	12 (9.4)	6 (11.8)	0.877
Diabetes mellitus	13 (2.61)	10 (3.1)	1 (0.8)	2 (3.9)	0.230
Marfan syndrome	7 (1.40)	5 (1.6)	2 (1.6)	0 (0.0)	1.000
History of cardiac surgical treatment	14 (2.81)	10 (3.1)	4 (3.1)	0 (0.0)	0.216
History of stroke	29 (5.81)	16 (5.0)	8 (6.3)	5 (9.8)	0.429
Peripheral vascular disease	6 (1.20)	4 (1.3)	1 (0.8)	1 (2.0)	0.666
Heart failure	18 (3.61)	13 (4.1)	2 (1.6)	3 (5.9)	0.251
COPD	4 (0.80)	2 (0.6)	2 (1.6)	0 (0.0)	0.561
Dyslipidemia	8 (1.60)	5 (1.6)	2 (1.6)	1 (2.0)	0.872
Massive pericardial effusion	10 (2.00)	6 (1.9)	4 (3.1)	0 (0.0)	0.247
Chronic kidney disease	13 (2.61)	8 (2.5)	4 (3.1)	1 (2.0)	0.890
WBC (10^9^/L)	10.59 (8.50–12.72)	10.61 (8.53–12.72)	10.45 (7.99–12.77)	11.30 (8.97–12.71)	0.650
HGB (g/L)	123.00 (109.00–135.00)	126.00 (110.00–136.00)	119.50 (108.00–134.00)	119.00 (108.00–134.00)	0.129
PLT (10^9^/L)	152.00 (123.00–190.50)	154.00 (126.25–192.75)	148.00 (120.00–187.50)	161.00 (122.00–200.00)	0.507
PT (s)	13.10 (12.10–14.40)	13.00 (12.10–14.30)	13.10 (12.00–14.80)	13.50 (12.40–14.60)	0.393
APTT (s)	30.80 (28.20–35.03)	30.50 (28.10–35.13)	30.80 (28.30–34.40)	31.30 (28.50–35.75)	0.684
DD (μg/ml)	2.51 (1.06–5.45)	2.44 (1.03–4.97)	2.41 (1.03–5.40)	4.33 (1.87–8.98)	0.078
ALT (U/L)	19.00 (13.00–34.00)	20.00 (13.00–36.25)	17.50 (12.00–27.00)	22.00 (13.00–37.00)	0.136
AST (U/L)	26.00 (17.00–52.00)	27.00 (17.00–52.00)	24.00 (16.00–61.00)	26.50 (19.00–52.25)	0.922
SCr (μmol/L)	84.00 (65.00–115.00)	85.00 (65.00–114.00)	84.00 (65.00–125.00)	80.00 (63.00–116.00)	0.475
CTn I (ng/L)	13.16 (2.52–333.64)	12.94 (2.42–288.93)	18.72 (2.95–719.08)	9.71 (1.50–365.35)	0.811
NT-proBNP	1057.00 (459.45–2242.50)	912.60 (512.60–1909.00)	1677.00 (519.13–3430.50)	908.55 (347.38–2596.50)	0.069
Time from symptom onset to be hospitalized (h)	15.00 (9.00–24.00)	15.00 (9.00–24.00)	14.50 (10.00–24.00)	12.00 (8.00–24.00)	0.770

Values are presented as the mean (SD), median (IQR), or number of patients (%).

COPD, chronic obstructive pulmonary disease; SD, standard deviation; IQR, interquartile range. WBC, white blood cell counts; HGB, hemoglobin; PLT, platelet; PT, prothrombin time; APTT, activated partial thromboplastin time; DD, D-Dimer; ALT, alanine aminotransferase; AST, aspartate aminotransferase; SCr, serum creatinine; CTn I, cardiac troponin I; n-terminal pro-brain natriuretic peptide, NT-proBNP.

### Comparison of weekdays, weekends and holidays

The baseline characteristics of the three groups are listed in Table 1. Among the 499 patients, 320 (64.13%) were in the weekday group, 128 (25.65%) were in the weekend group and 51 (10.22%) were in the holiday group. There was no significant difference in age, sex, comorbidity, laboratory examination or the time from the onset of symptoms to admission among the three groups.

As shown in Table 2, there was no significant difference among the three groups in terms of CPB time, operation time, intraoperative blood transfusion, concomitant operation, time from admission to operation intervention, ICU stay, hospital stay, postoperative complications, 7-day mortality or in-hospital mortality. However, there was a significant difference in the number of patients undergoing daytime and nighttime surgery among the three groups (*P* < 0.001). The proportion of nighttime operations was higher in the weekday group [66.2% (*n* = 212/320)], while the proportion of daytime operations was higher in the weekend group and the holiday group [62.5% (*n* = 80/128) and 76.5% (*n* = 39/51), respectively].

**TABLE 2:** Intraoperative and postoperative patient characteristics.

Variable	Total cohort (*n* = 499)	Weekday (*n* = 320)	Weekend (*n* = 128)	Holiday (*n* = 51)	*P*-value
CPB time (min)	221.50 (191.25–267.00)	225.50 (192.00–268.50)	224.00 (191.50–271.25)	207.50 (184.74–255.25)	0.300
Operation time (min)	505.00 (440.00–575.00)	505.00 (445.00–578.75)	510.00 (440.00–580.00)	460.00 (430.00–560.00)	0.134
Autologous blood transfusion (ml)	573.69 (500.00–573.69)	573.69 (500.00–573.69)	573.69 (500.00–573.69)	573.69 (500.00–573.69)	0.785
PRBC transfusion (U)	4.00 (2.00–4.20)	4.00 (2.00–4.20)	4.00 (2.00–4.20)	4.00 (2.00–4.20)	0.496
Plasma transfusion (mL)	500.00 (400.00–600.00)	500.00 (400.00–647.50)	500.00 (400.00–600.00)	400.00 (400.00–650.00)	0.462
Cryoprecipitate transfusion (U)	17.56 (16.00–17.56)	17.56 (16.00–17.56)	17.56 (16.00–17.56)	17.56 (16.00–17.56)	0.923
Platelet transfusion (U)	2.00 (1.96–2.00)	2.00 (1.96–2.00)	2.00 (1.96–2.00)	2.00 (1.96–2.00)	0.735
**Concomitant operation**					
Bentall	64 (12.83)	37 (11.6)	21 (16.4)	6 (11.8)	0.389
CABG	22 (4.41)	17 (5.3)	5 (3.9)	0 (0.0)	0.109
MVR	4 (0.80)	1 (0.3)	3 (2.3)	0 (0.0)	0.108
Pneumonia	73 (14.6)	44 (13.8)	21 (16.4)	8 (15.7)	0.747
Tracheotomy	27 (5.4)	18 (5.6)	6 (4.7)	3 (5.9)	0.956
Acute kidney injury	146 (29.3)	98 (30.7)	35 (27.3)	13 (25.5)	0.654
CRRT	59 (11.8)	39 (12.2)	14 (10.9)	6 (11.8)	0.955
Stroke	24 (4.8)	13 (4.1)	8 (6.3)	3 (5.9)	0.668
Hepatic hypofunction	105 (21.1)	63 (19.7)	31 (24.4)	11 (21.6)	0.538
Gastrointestinal bleeding	23 (4.6)	23 (3.8)	8 (6.3)	3 (5.9)	0.496
Multisystem organ failure	42 (8.4)	23 (7.2)	15 (11.7)	4 (7.8)	0.319
**Surgical intervention time (T)**					0.096
T ≤ 48 h	221 (44.3)	142 (64.3)	50 (22.6)	29 (13.1)	
T > 48 h	278 (55.7)	178 (64.0)	78 (28.1)	22 (7.9)	
**Daytime or Nighttime**					0.001
Daytime	227 (45.5)	108 (33.8)	80 (62.5)	39 (76.5)	
Nighttime	272 (54.5)	212 (66.2)	48 (37.5)	12 (23.5)	
**Clinical outcomes**					0.740
Cure	380 (76.2)	248 (77.5)	93 (72.7)	39 (76.5)	
Automatic discharge	69 (13.8)	44 (13.8)	19 (14.8)	6 (11.8)	
Death	50 (10.0)	28 (8.8)	16 (12.5)	6 (11.8)	
ICU stay (d)	5.00 (3.00–7.00)	5.00 (3.00–7.00)	5.00 (3.00–7.00)	5.00 (4.00–7.00)	0.826
Hospital stay (d)	19.00 (14.00–23.00)	19.00 (14.00–23.75)	19.00 (14.00–23.00)	19.00 (15.00–24.00)	0.694
7-day mortality	28 (5.6)	15 (4.7)	10 (7.8)	3 (5.9)	0.515
In-hospital mortality	50 (10.0)	28 (8.8)	16 (12.5)	6 (11.8)	0.451

Bentall, aortic valve replacement with a valved conduit; CABG, coronary artery bypass grafting; MVR, mitral valve replacement; CPB, cardiopulmonary bypass; PRBC, packed red blood cell; CRRT, continuous renal replacement therapy.

### Comparison of daytime and nighttime operations

According to the start and end time of surgery, 227 (45.5%) of 499 patients underwent surgery during the daytime, while 272 (54.5%) underwent surgery at nighttime. As shown in Table 3, there was no significant difference in the baseline characteristics between the daytime group and the nighttime group. Compared with the daytime group, the D-dimer (DD) [2.99 (1.20–5.95) vs. 2.09 (0.94–4.84), *P* = 0.006] and Aspartate aminotransferase (AST) [29.00 (18.25–59.75) vs. 23.00 (16.00–46.00), *P* = 0.005] in the nighttime group were significantly higher (*P* < 0.01), and the n-terminal pro-brain natriuretic peptide (NT-proBNP) [794.95 (436.08–1569.69) vs. 1727.00 (645.20–3317.00), *P* = 0.001] was significantly lower –(*P* < 0.01), with statistical difference.

**TABLE 3:** Preoperative characteristics of daytime and nighttime groups or intervention times ≤48 h and >48 h.

Variable	Daytime (*n* = 227)	Nighttime (*n* = 272)	*P*-value	Intervention time ≤ 48 h (*n* = 221)	Intervention time > 48 h (*n* = 278)	*P*-value
Age (years)	53.04 (11.69)	52.97 (12.09)	0.949	52.01 (11.93)	53.78 (11.83)	0.098
Sex (male)	163 (71.8)	189 (69.5)	0.571	160 (72.4)	192 (69.1)	0.417
Hypertension	180(79.3)	208 (76.5)	0.450	177 (80.1)	211 (75.9)	0.263
Coronary disease	27 (11.9)	25 (9.2)	0.325	20 (9.0)	32 (11.5)	0.371
Diabetes mellitus	5 (2.2)	8 (3.0)	0.601	5 (2.3)	8 (2.9)	0.664
Marfan syndrome	4 (1.8)	3 (1.1)	0.809	1 (0.45)	7 (2.52)	0.068
History of cardiac surgical treatment	5 (2.2)	9 (3.3)	0.456	6 (2.7)	8 (2.9)	0.913
History of stroke	16 (7.0)	13 (4.8)	0.281	11 (5.0)	18 (6.5)	0.478
Peripheral vascular disease	2 (0.9)	4 (1.5)	0.850	3 (1.4)	3 (1.1)	1.000
Heart failure	9 (4.0)	9 (3.3)	0.696	9 (4.1)	9 (3.2)	0.619
COPD	2 (0.9)	2 (0.7)	1.000	1 (0.5)	3 (1.1)	0.633
Dyslipidemia	5 (2.2)	3 (1.1)	0.538	4 (1.8)	4 (1.4)	0.737
Massive pericardial effusion	1 (0.4)	9 (3.3)	0.050	3 (1.4)	7 (2.5)	0.524
Chronic kidney disease	8 (3.5)	5 (1.8)	0.239	4 (1.8)	9 (3.2)	0.320
WBC (10^9^/L)	10.40 (8.06–12.60)	10.79 (8.79–12.81)	0.650	11.60 (9.33–13.42)	10.11 (9.37–13.41)	0.001
HGB (g/L)	122.00 (111.00–134.00)	124.00 (108.00–138.00)	0.330	127.00 (110.00–139.00)	121.00 (108.00–133.00)	0.004
PLT (10^9^/L)	158.50 (124.25–196.75)	146.50 (122.00–185.75)	0.146	147.00 (120.00–184.00)	158.00 (127.00–200.00)	0.038
PT (s)	13.20 (12.10–14.40)	13.10 (12.10–14.43)	0.926	13.30 (12.20–14.60)	13.00 (12.10–14.20)	0.149
APTT (s)	30.20 (28.10–34.15)	31.30 (28.30–35.83)	0.056	32.0 (28.90–36.60)	29.70 (27.80–33.35)	0.001
DD (μg/ml)	2.09 (0.94–4.84)	2.99 (1.20–5.95)	0.006	4.33 (2.36–11.59)	1.56 (0.79–2.94)	0.001
ALT (U/L)	18.00 (12.00–30.00)	21.00 (13.00–37.00)	0.057	22.00 (14.00–39.50)	17.00 (11.25–29.75)	0.002
AST (U/L)	23.00 (16.00–46.00)	29.0 (18.25–59.75)	0.005	35.00 (22.00–76.00)	21.00 (15.00–38.00)	0.001
SCr (μmol/L)	80.00 (63.00–115.00)	89.00 (66.00–115)	0.175	90.00 (69.00–124.00)	80.00 (63.00–106.75)	0.002
CTn I (ng/L)	10.10 (2.63–332.82)	19.74 (2.33–341.25)	0.651	24.67 (2.88–445.87)	10.24 (2.04–264.04)	0.317
NT-proBNP	1727.00 (645.30–3317.00)	794.95 (436.08–1569.69)	0.001	909.20 (520.70–1732.00)	1245.50 (482.13–2672.25)	0.337
Time from symptom onset to be hospitalized (h)	15.00 (10.00–24.00)	14.00 (9.00–24.00)	0.165	10.00 (7.00–17.00)	24.00 (12.00–48.00)	0.001

As shown in Table 4, there was no significant difference between the daytime group and the nighttime group in terms of operation time, intraoperative autologous blood, plasma and cryoprecipitate transfusion, concomitant surgery, postoperative complications, hospitalization outcome, etc. Compared with the daytime group, the 7-day mortality rate [6.6% (*n* = 18/272) vs. 4.4% (*n* = 10/227)] and in-hospital mortality rate [10.7% (*n* = 29/272) vs. 9.2% (*n* = 21/227)] in the nighttime group were higher, but there was no significant difference. There were significant differences between the two groups in CPB time, intraoperative packed red blood cell (PRBC) and platelet transfusion, ICU stay and hospital stay (*P* < 0.05). Compared with the daytime group, the nighttime group had a longer CPB time [230.00 (195.00–275.00) vs. 214.00 (188.00–257.00), *P* < 0.01], more intraoperative PRBC transfusions [4.00 (3.25–4.20) vs. 4.00(2.00–4.20), *P* < 0.05] and fewer platelet transfusions [1.98 (1.96–2.00) vs. 2.00 (1.96–2.00), *P* < 0.01]. However, in terms of ICU stay [4.00 (3.00–7.00) vs. 5.00 (4.00–7.00), *P* < 0.05] and hospital stay [18.00 (14.00–22.00) vs. 20.00 (15.00–25.00), *P* < 0.01], the nighttime group was shorter, with a significant difference.

**TABLE 4:** Intraoperative and postoperative characteristics of daytime and nighttime groups or intervention times ≤48 H and >48 H.

Variable	Daytime (*n* = 227)	Nighttime (*n* = 272)	*P*-value	Intervention time ≤ 48 h (*n* = 221)	Intervention time > 48 h (*n* = 278)	*P*-value
CPB time (min)	214.00 (188.00–257.00)	230.00 (195.00–275.00)	0.007	226.00 (195.25–269.75)	216.00 (189.25–266.75)	0.247
Operation time (min)	505.00 (445.00–570.00)	502.50 (440.00–580.00)	0.680	505.00 (445.00–580.00)	500.00 (440.00–575.00)	0.429
Autologous blood transfusion (mL)	573.69 (400.00–573.69)	573.69 (500.00–573.69)	0.932	573.69 (500.00–573.69)	573.69 (400.00–573.69)	0.054
PRBC transfusion (U)	4.00 (2.00–4.20)	4.00 (3.25–4.20)	0.041	4.00 (2.00–5.50)	4.00 (2.00–4.00)	0.058
Plasma transfusion (mL)	500.00 (400.00–600.00)	500.00 (400.00–615.00)	0.288	500.00 (400.00–725.00)	500.00 (400.00–600.00)	0.345
Cryoprecipitate transfusion (U)	17.56 (16.00–17.56)	17.56 (16.00–17.56)	0.966	17.56 (16.00–17.56)	17.56 (16.00–17.56)	0.800
Platelet transfusion (U)	2.00 (1.96–2.00)	1.98 (1.96–2.00)	0.008	2.00 (1.96–2.00)	2.00 (1.96–2.00)	0.525
**Concomitant operation**						
Bentall	30 (13.2)	34 (12.5)	0.893	29(13.1)	35(12.6)	0.860
CABG	10 (4.4)	12 (4.4)	1.000	7 (3.2)	15 (5.4)	0.228
MVR	2 (0.9)	2 (0.7)	1.000	2 (0.9)	2 (0.7)	1.000
Pneumonia	35 (15.4)	38 (14.0)	0.703	31 (14.0)	42 (15.1)	0.734
Tracheotomy	14 (6.2)	13 (4.8)	0.554	12 (5.4)	15(5.4)	0.987
Acute kidney injury	70 (30.8)	76 (28.0)	0.553	54 (24.4)	92 (33.1)	0.035
CRRT	30 (13.2)	29 (10.7)	0.406	24 (10.9)	35 (12.6)	0.552
Stroke	9 (4.0)	15 (5.0)	0.530	14 (6.3)	10 (3.6)	0.156
Hepatic hypofunction	51 (22.6)	54 (19.9)	0.508	48 (21.7)	57 (20.6)	0.756
Gastrointestinal bleeding	7 (3.1)	16(5.9)	0.198	13 (5.9)	10 (3.6)	0.222
Multisystem organ failure	25 (11.0)	17 (6.3)	0.074	18 (8.1)	24 (8.6)	0.845
Clinical outcomes			0.531			0.639
Cure	179 (78.9)	203 (74.6)		173 (78.3)	212 (76.3)	
Automatic discharge	27 (11.9)	40 (14.7)		29 (13.1)	35 (12.6)	
Death	21 (9.2)	29 (10.7)		19 (8.6)	31 (11.1)	
ICU stay (d)	5.00 (4.00–7.00)	4.00 (3.00–7.00)	0.010	4.00 (3.00–7.00)	5.00 (4.00–7.00)	0.017
Hospital stay (d)	20.00 (15.00–25.00)	18.00 (14.00–22.00)	0.007	18.00 (14.00–22.00)	20.00 (15.00–24.25)	0.018
7-day mortality	10 (4.4)	18 (6.6)	0.332	11 (5.0)	17(6.1)	0.583
In-hospital mortality	21 (9.2)	29 (10.7)	0.601	19 (8.6)	31 (11.1)	0.345

### Comparison of surgical intervention times ≤48 h and >48 h

With reference to the 48-h intervention time, 221 (44.3%) patients underwent surgery within 48 h of the onset of symptoms, and 278 (55.7%) patients underwent surgical intervention for more than 48 h. There was no significant difference between the two groups in terms of age, sex or comorbidity (Table 3). Compared with the group with intervention time ≤48 h, the white blood cell counts (WBC) (10.11 [9.37–13.41] vs. 11.60 [9.33–13.42], *P* = 0.001), hemoglobin (HGB) (121.00[108.00–133.00]) vs. 127.00 [110.00–139.00], *P* = 0.004), activated partial thromboplastin time (APTT) (29.70 [27.80–33.35]) vs. 32.00 [28.90–36.60], *P* = 0.001), DD (1.56 [0.79–2.94] vs. 4.33 [2.36–11.59]), *P* = 0.001), alanine aminotransferase (ALT) (17.00 [11.25–29.75]) vs. 22.00 [14.00–39.50], *P* = 0.002), AST (21.00 [15.00–38.00] vs. 35.00 [22.00–76.00]), *P* = 0.001) and serum creatinine (SCr) (80.00 [63.00–106.75] vs. 90.00 [69.00–124.00]), *P* = 0.002) were significantly decreased in the preoperative laboratory examination of the >48 h group, but the median values of the above indicators were within the normal range. The platelet (PLT) of >48 h group was significantly higher than that of ≤48 h group (158.00 [127.00–20.00]) vs. 147.00 [120.00–184.00], *P* = 0.038).

As shown in Table 4, there was no significant difference between the two groups in all intraoperative and postoperative characteristics, except for postoperative acute renal injury, ICU stay and hospital stay. The 7-day mortality rate [6.1% (*n* = 17/278) vs. 5.0% (*n* = 11/221), *P* = 0.583] and in-hospital mortality rate [11.1% (*n* = 31/278) vs. 8.6% (*n* = 19/221), *P* = 0.345] in the intervention time >48 h group were higher than those in the intervention time ≤48 h group, but there was no significant difference. In addition, the incidence of postoperative acute renal injury [33.1% (*n* = 92/278) vs. 24.4% (*n* = 54/221), *P* = 0.035], ICU stay [5.00 (4.00–7.00) vs. 4.00 (3.00–7.00), *P* = 0.017] and hospital stay [20.00 (15.00–24.25) vs. 18.00 (14.00–22.00), *P* = 0.018] in the intervention time >48 h group were significantly higher than those in the intervention time ≤48 h group, with significant differences.

### Comparison of working hours and off- hours groups

Four hundred ninety-nine patients were divided into two groups according to working hours and off-hours. Among them, 108 patients underwent surgery during working hours and 391 patients underwent surgery during off-hours. We analyzed the intraoperative and postoperative characteristics of the two groups. There were significant differences between the two groups only in platelet transfusion [2.00 (1.96–2.00) vs. 2.00 (1.96–2.00), *P* = 0.017], the incidence of gastrointestinal bleeding after surgery [0.9% (*n* = 1/108) vs. 5.9% (*n* = 22/391), *P* = 0.038] and ICU stay [5.00(4.00–8.00) vs. 5.00(3.00–7.00), *P* = 0.011] (Table 5).

**TABLE 5:** Intraoperative and postoperative characteristics of working hours and off- hours groups.

Variable	Working hours(*n* = 108)	Off-hours (*n* = 391)	*P-*value
CPB time (min)	215.00 (187.75–262.25)	225.00 (193.75–270.25)	0.107
Operation time (min)	520.00 (460.00–578.75)	500.00 (440.00–575.00)	0.125
Autologous blood transfusion (ml)	573.69 (500.00–573.69)	573.69 (500.00–573.69)	0.381
PRBC transfusion (U)	4.00 (2.00–4.20)	4.00 (2.00–4.20)	0.171
Plasma transfusion (mL)	534.10 (400.00–650.00)	500.00 (400.00–600.00)	0.761
Cryoprecipitate transfusion (U)	17.56 (16.00–17.56)	17.56 (16.00–17.56)	0.834
Platelet transfusion (U)	2.00 (1.96–2.00)	2.00 (1.96–2.00)	0.017
**Concomitant operation**			
Bentall	12 (11.1)	52 (13.3)	0.547
CABG	7 (6.5)	15 (3.8)	0.286
MVR	0 (0.0)	4 (1.0)	0.582
Pneumonia	16 (14.8)	57 (14.6)	0.951
Tracheotomy	8 (7.4)	19 (4.9)	0.300
Acute kidney injury	39 (36.1)	107 (27.4)	0.080
CRRT	18 (16.7)	41 (10.5)	0.080
Stroke	3 (2.8)	21 (5.4)	0.265
Hepatic hypofunction	23 (21.3)	82(21.0)	0.951
Gastrointestinal bleeding	1 (0.9)	22 (5.9)	0.038
Multisystem organ failure	10 (9.3)	32 (8.2)	0.722
**Clinical outcomes**			0.623
Cure	84 (77.8)	296 (75.7)	
Automatic discharge	12 (11.1)	57 (14.6)	
Death	12 (11.1)	38 (9.7)	
ICU stay (d)	5.00 (4.00–8.00)	5.00 (3.00–7.00)	0.011
Hospital stay (d)	21.00 (15.00–25.00)	18.00 (14.00–23.00)	0.059
7-day mortality	2 (1.9)	26 (6.6)	0.059
In-hospital mortality	12 (11.1)	38 (9.7)	0.670

### Comparison of the 7-day and in-hospital mortality of patients in different years

We compared the 7-day and in-hospital mortality of patients in different years. In order to balance the number of patients between different years, they were divided into one groups every two years. As shown in Table 6, there is no significant difference in 7-day mortality and in-hospital mortality among patients in different years.

**TABLE 6:** The 7-day and in-hospital mortality of patients in different years.

Variable	2015.01–2016.12 (*n* = 134)	2017.01–2018.12 (*n* = 163)	2019.01–2020.12 (*n* = 107)	2021.01–2022.03 (*n* = 95)	*P*-value
7-day mortality	13 (9.7)	6 (3.7)	4 (3.7)	5(5.3)	0.106
In-hospital mortality	16 (11.9)	13 (8.0)	12 (11.2)	9 (9.5)	0.681

### Survival analysis

**FIGURE 3:**
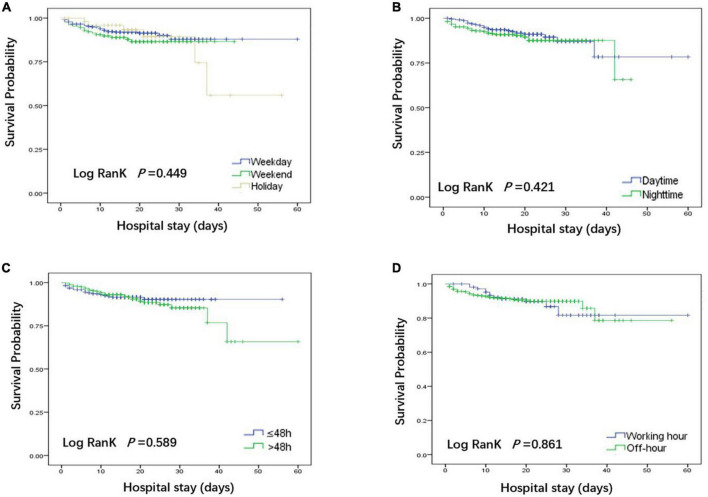
Kaplan-Meier curves were used to compare the in-hospital deaths of patients in each group. **(A)** Comparison of patients undergoing weekdays, weekends and holidays; **(B)** Comparison of patients undergoing daytime and nighttime surgery; **(C)** Comparison of patients with surgical intervention times ≤48 h and >48 h. **(D)** Comparison of patients undergoing working hours and off-hours.

The log-rank test was used to test the differences between groups, as shown in Figure 3. There was no significant difference in the in-hospital mortality among the weekday group, weekend group and holiday group, daytime group and nighttime group, intervention time ≤48 h group and >48 h group, working hours group and off-hours group.

Cox regression analysis was conducted to study the correlation between 7-day death and in-hospital death and various factors. First, univariate Cox regression analysis was performed on each variable to screen out the variables with *P* < 0.1. After using a bivariate correlation test to exclude confounding factors, the remaining eligible variables were included in multivariate Cox regression.

The univariate Cox regression analysis found that the HR value and 95% CI of each variable in the preoperative laboratory examination were very close to 1, suggesting that it was not related to the 7-day and hospital death, so it was not included in the multivariate analysis. As shown in Table 7, multivariate Cox regression analysis showed that different groups had no effect on the 7-day mortality and in-hospital mortality (*P* > 0.05). The risk factors of 7-day death and in-hospital death included CPB time (HR = 1.010, 95% CI 1.006–1.014, *P* = 0.001) (HR = 1.007, 95% CI 1.004–1.011, *P* = 0.001), intraoperative PRBC blood transfusion (HR = 1.210, 95% CI 1.094–1.338, *P* = 0.001) (HR = 1.122, 95% CI 1.029–1.223, *P* = 0.009), postoperative pneumonia (HR = 5.298, 95% CI 1.437–19.534, *P* = 0.012) (HR = 2.542, 95% CI 1.186–5.450, *P* = 0.016), acute renal function injury (HR = 3.085, 95% CI 1.302–7.310, *P* = 0.011) (HR = 2.423, 95% CI 1.214–4.834, *P* = 0.012) and multiple system organ failure (HR = 6.041, 95% CI 2.400–15.206, *P* = 0.001) (HR = 11.200, 95% CI 5.549–22.605, *P* = 0.001), with statistical significance.

**TABLE 7:** Risk analysis of 7-day death and in-hospital death.

	Deaths within 7 days				Deaths within hospital stay			
Risk factor	B	HR	95% CI	*P-*value	B	HR	95% CI	*P-*value
CPB time	0.010	1.010	1.006–1.014	0.001	0.007	1.007	1.004–1.011	0.001
PRBC transfusion	0.190	1.210	1.094–1.338	0.001	0.115	1.122	1.029–1.223	0.009
Acute kidney injury	1.126	3.085	1.302–7.310	0.011	0.855	2.423	1.214–4.834	0.012
Multisystem organ failure	1.799	6.041	2.400–15.206	0.001	2.416	11.200	5.549–22.605	0.001
Pneumonia	1.667	5.298	1.437–19.534	0.012	0.933	2.542	1.186–5.450	0.016
Weekday/Weekend/Holiday	0.301	1.352	0.791–2.309	0.270	0.136	1.146	0.766–1.716	0.508
Daytime/Nighttime	0.430	1.538	0.657–3.597	0.321	–0.193	0.825	0.450–1.514	0.534
≤48h/>48 h	–0.572	0.564	0.256–1.242	0.155	–0.333	0.717	0.404–1.271	0.717
Working hours/Off-hours	1.307	3.695	0.877–15.570	0.075	–0.58	0.944	0.493–1.807	0.861

## Discussion

Recent reports from large datasets show that the operative mortality after ATAAD repair ranges from 17 to 20% (15). Data from a Beijing Heart Surgery Center in China obtained for 10 years showed that the operative mortality rate of ATAAD was 7.5% (16). Our results showed that the in-hospital mortality of ATAAD patients within 7 days of symptom onset was 10.0%.

Studies have confirmed that weekend or holiday effects will increase inpatient mortality (9, 10, 17, 18). For ATAAD patients, nighttime surgery may also be an independent high-risk factor for in-hospital death (7). Due to the imbalance of medical resources and economic conditions in China, most ATAAD patients need to be transferred to tertiary hospitals or heart centers for surgery. The latest research showed that in China, the mean interval from symptom onset to surgery for ATAAD was 5 days (19). Our results showed that there was no difference in inpatient mortality, 7-day mortality and postoperative complications among patients on weekdays, weekends or holidays, as well as during the daytime or nighttime, provided that the emergency green channel integrated management strategy and the specialized cardiac operative teams had dedicated nighttime, weekend or holiday shifts. This was consistent with the recent research results of IRAD (9). In addition, our cohort analysis of working hours and off-hours showed that there was also no difference between 7-day mortality and in-hospital mortality.

We also found that although there was no difference in mortality, the CPB time of the nighttime group was longer than that of the daytime group, but it did not affect the operation time, which may be related to the fatigue of doctors at night. Early studies have shown that cardiac surgeons, anesthesiologists and perfusionists will be in a state of fatigue during off-hours (20). The difference in PRBC and platelet input between the two groups may be related to the use of mean substitution of missing values, which has no clinical significance. The difference in ICU stay and hospital stay between the two groups may be related to our time unit (d).

Studies have shown that delaying the time of surgical intervention can increase the risk of death in ATAAD patients (1, 5). To more reliably describe the survival rate after aortic dissection, IRAD proposed a new time classification of aortic dissection in 2013: hyperacute (0–24 h), acute (2–7 days), subacute (8–30 days), and chronic (≥30 days) (13). Considering the high time-related mortality of ATAAD, we followed the new classification of IRAD and only included patients diagnosed with ATAAD from the onset of symptoms to 7 days. This study showed that compared with the intervention time ≤48 h group, there was no difference in 7-day mortality and in-hospital mortality in the intervention time >48 h group, but the incidence of postoperative acute renal injury was significantly higher in the group with more than 48 h of intervention, accompanied by a significant extension of ICU stay and hospital stay.

The fatigue of doctors may be closely related to the prognosis of patients. Doctor fatigue may increase the operation time, cardiopulmonary bypass time and aortic occlusion time. A longer cardiopulmonary bypass time is also considered to be a high-risk factor for acute renal injury, which further leads to higher in-hospital mortality (21). Our results showed that there was no difference in CPB time or operation time between the group with an intervention time >48 h and the group with an intervention time ≤48 h. The operation team adopted the form of shift and requires team members to ensure at least 3 h of rest before operation, so it can also be explained that this working mode helps to improve the fatigue and poor performance of team members. On the other hand, the 7-day mortality and in-hospital mortality of the group with intervention time >48 h did not increase, indicating that rapid and effective control of blood pressure, pulse and pain can help stabilize the patient’s condition and reduce the patient’s mortality after the patient is admitted to the hospital.

Clinical studies have shown that acute renal function injury is a common complication after ATAAD, with an incidence of approximately 40.6%, which can independently predict poor long-term prognosis (22). Our study confirmed that the extension of intervention time will increase the incidence of postoperative acute renal function injury. Multivariate Cox regression showed that postoperative acute renal function injury was an independent risk factor for hospitalized death in admitted patients, suggesting that we need early prevention and intervention of perioperative renal function.

Although there may be significant differences in preoperative laboratory test results between different groups, univariate Cox regression found that these factors had little effect on 7-day and in-hospital death. Our multivariate Cox regression also found that intraoperative CPB time, intraoperative PRBC transfusion, postoperative acute kidney injury, pneumonia and multiple organ dysfunction were independent risk factors for 7-day and in-hospital death. There are significant differences in intraoperative PRBC transfusion, which may be related to the application of intraoperative blood management technology. The increase in intraoperative CPB time is a predictor of the immediate incidence rate and mortality after adult heart surgery (23). Hemolysis may occur during CPB and is associated with acute renal injury after surgery (24). In addition to acute renal insufficiency, ATAAD patients are also prone to multiple organ dysfunction, such as pneumonia, liver dysfunction, coagulation disorder, gastrointestinal bleeding, and stroke, which is related to in-hospital mortality (14, 25). Therefore, to improve the postoperative recovery quality and survival rate of ATAAD patients, we should actively avoid or treat postoperative complications.

The latest large-scale cross-sectional study confirmed that there was no link between surgeon fatigue caused by overnight surgery and adverse perioperative outcomes of patients (26). However, it is well known that compared with the general surgical population, the risk of heart and aortic surgery and the incidence of postoperative adverse events are higher. ATAAD emergency surgery patients are more serious, and the operation is more difficult and takes longer, which requires the cooperation and coordination of the intraoperative team. Therefore, further research is needed to confirm whether the occupational fatigue of team medical staff caused by night or long-term surgery affects the perioperative survival rate of emergency surgery patients with ATAAD.

This study has some limitations. (1) This is a retrospective study, and the results may be affected by the non-randomized nature. (2) This study only included patients who underwent aortic arch replacement surgery, not patients who died before and during surgery, and we excluded patients who had symptoms for more than 7 days. The results could not be extended to all ATAAD patients. (3) Our ICU stay was not calculated by the hour, which had a large error and may affect the results. (4) The patients did not receive follow-up after discharge; it was impossible to obtain more credible and accurate conclusions.

## Conclusion

Patients with ATAAD should be operated on as soon as the diagnosis is confirmed. If the operation is delayed for special reasons after admission, the patient should be given rapid and effective control of blood pressure, pulse and pain. Postoperative acute renal function injury, pneumonia and multiple organ failure were the main risk factor for 7-day death and in-hospital death. Under the premise of the emergency green channel integrated management strategy and the shift of a special cardiac surgery team at nighttime, weekends or holidays, the surgery time was not related to the in-hospital mortality or 7-day mortality of patients.

## Data availability statement

The raw data supporting the conclusions of this article will be made available by the authors, without undue reservation.

## Ethics statement

The studies involving human participants were reviewed and approved by Qilu Hospital of Shandong University Medical Ethics Committee Shandong University. Written informed consent for participation was not required for this study in accordance with the national legislation and the institutional requirements.

## Author contributions

XZ, KL, XL, and WL: data collection. SY, XZ, and KL: statistical analysis. SY and KL: writing of the manuscript. SY and XL: inception of the study idea. SY, KL, and WL: study design and revision of the manuscript. All authors approved the final manuscript.
